# COVID-19 preventive behaviours in White British and Black, Asian and Minority Ethnic (BAME) people in the UK

**DOI:** 10.1177/13591053211017208

**Published:** 2021-05-15

**Authors:** Glynis M Breakwell, Emanuele Fino, Rusi Jaspal

**Affiliations:** 1 University of Bath, UK; 2 Imperial College London, UK; 3 Nottingham Trent University, UK

**Keywords:** COVID-19 prevention, COVID-19 risk, ethnicity, ingroup power, trust in science

## Abstract

A model of the effects of ethnicity, political trust, trust in science, perceived ingroup power, COVID-19 risk and fear of COVID-19 upon likelihood of COVID-19 preventive behaviour (CPB) is presented. The structural equation model was a good fit for survey data from 478 White British and Black, Asian and Minority Ethnic (BAME) people. Ethnicity had a direct effect on CPB (BAME reported higher CPB) and an indirect effect on it through political trust, ingroup power, COVID-19 risk and trust in science. Ethnicity was not significantly related to COVID-19 fear. COVID-19 fear and trust in science were positively associated with CPB.

## Introduction

COVID-19 has developed into a global pandemic with high morbidity and mortality rates. Initial attempts to contain the spread of the virus in the United Kingdom (UK) via widespread curtailment of interpersonal contact (labelled ‘lockdown’ or, for those at highest risk, ‘shielding’) between March and July 2020 proved effective but when containment measures were eased infection rates rose steeply. By September 2020, the UK government had introduced renewed restrictions regionally. This proved inadequate to stem the increasing rate of infection. A second national lockdown was introduced in November 2020 and, after a brief respite over Christmas, was renewed in January 2021 as a new variant of the virus was discovered in the UK. The government’s preventive approach, prior to the authorisation of the use of newly developed vaccines in December 2020, entailed social distancing that included mandatory wearing of face masks in specific contexts, limiting of contacts outside of one’s household, home-working and testing and tracing methods (Breakwell et al., 2021; Michie et al., 2020).

The study reported here examines some factors that predict the likelihood that an individual will comply with official guidelines on preventive behaviours. We particularly investigate ethnic differences in patterns of COVID-19 preventive behaviour and the social psychological factors associated with them. To do so seems especially relevant since there is evidence that people from Black, Asian and Minority Ethnic (BAME) groups are disproportionately affected by COVID-19 (Sze et al., 2020). In the UK, BAME is an acronym used for people who are of Black, Asian, or minority ethnicity and is used as a demographic category (Alexander, 1999; Aspinall, 2002). BAME is regarded as useful for describing collective experiences (see Wellcome, 2020), and is commonly used in the public sector and across higher education. However, use of the BAME term has faced criticism because it includes, and treats as homogeneous, groups that vary in educational and occupational opportunity and achievement (Strand, 2015), and are very diverse in terms of ethnicity, culture, language, religion and history. Also, within the BAME category, differences in ethnic identification and ‘Britishness’ have been reported (Jaspal et al., 2020). Despite the evident diversity of its membership, the BAME categorisation has social meaning. It has acquired the status of a ‘conceptual group’ (i.e. a categorisation imposed on people by a powerful source for its own purposes, Breakwell, 1979). BAME people now do use it as a self-descriptor (often in intergroup contexts) and may claim (and sometimes reject) identification with it. As a conceptual group, it can influence member cognition and action, besides changing the treatment of members by non-members.

### BAME compared with White British people in COVID-19 reactions

UK COVID-19 incidence reports (ONS, 2020) record higher rates of infection and fatality in BAME than in White British people. While no medical explanation for the difference in infection has been established, it may be in part explained by differentials in socio-economic status, living conditions and educational attainment (Bentley, 2020) or greater occupational exposure to the virus (since BAME are disproportionately represented in the health and care services workforce, Chaudry et al., 2020). In addition, the difference between BAME and White British people specifically in rates of infection, rather than severity of the illness once infected, may be associated with variations in preventive behaviour (that may be themselves linked to life circumstances, Nettle, 2010). Wellcome (2020) found that BAME people were more likely than White people to find it difficult to follow restrictions put in place by the government (50% vs 38%) and that they were less likely to say that information about coronavirus was very clear (52% vs 71%). This may affect preventive behaviour patterns. For instance, a higher percentage of BAME than White British people are reported to have said they would not take a COVID-19 vaccine (Robertson et al., 2021). Our study specifically examines differences between BAME and White British people in their self-reported likelihood of engaging in COVID-19 preventive behaviours.

COVID-19 and its social, economic and psychological sequelae have damaged not only the physical but also the mental health of the general UK population (Lopes et al., 2020; Rajkumar, 2020). ‘Compared with White British, BAME people are at greater risk of morbidity and mortality associated with COVID-19, but also of poor mental health outcomes during the pandemic’. Perceived inequalities of treatment during the coronavirus outbreak may be influencing this. Jaspal and Lopes (2021) found that, when people categorised as BAME have decreased identification with relevant social groups (e.g. the nation, ethnicity, religion) or perceive themselves to be discriminated against due to their ethnicity, they experience greater fear of COVID-19 and poorer mental health. There was a positive correlation between discrimination and fear of COVID-19.

### Factors predicting preventive behaviour

Likelihood of COVID-19 preventive behaviour (CPB) is affected by many factors. These coalesce around whether the person knows what to do, feels capable of doing it, and thinks it compatible with personal needs, habits, values and beliefs. Research has focussed on the impact of three main factors upon CPB: perceived own risk of COVID-19 infection; fear of COVID-19; and, awareness of CPB guidance and trust in the source of that guidance.

#### Perceived own risk

Perceived own risk in relation to health hazards influences behaviour (Clifton et al., 2016; Kahle et al., 2018). Yıldırım et al. (2020) found that perceived risk of COVID-19 was a significant predictor of preventive behaviour. Despite the pervasive social representations of the risk and severity of COVID-19, there is still variation in how individuals perceive their own risk. Individual risk estimates can be influenced by socio-demographic characteristics, past experience, personality traits, emotional state, ideological and belief systems, identity processes, and many other factors (Breakwell, 2014a).

#### Fear of COVID-19

Jaspal et al. (2020) argued that it is important to differentiate between fear of COVID-19, which refers to the affective state triggered in relation to COVID-19, and perceived own risk of contracting the disease. Nevertheless, they found that own risk and fear are correlated, with perceived risk heightening fear. Preventive behaviour can also be stimulated by being generally fearful or becoming afraid in a particular situation (Fischhoff et al., 2005; Weinstein et al., 2000). ‘Functional’ fear has been shown to be an adaptive response to COVID-19 associated with preventive behaviours (Harper et al., 2020).

#### Trust and ingroup power

During the COVID-19 pandemic, often-competing social representations of severity, risk and preventive behaviours have proliferated (Georgiou et al., 2020; Krause et al., 2020). Social representations of new illnesses influence reactions to health guidance (Joffe and Lee, 2004). Complex conspiracy theories about the origin of COVID-19 and the motives behind the introduction of behavioural restrictions (Jolley and Paterson, 2020) have fostered much uncertainty and mistrust. The competence and trustworthiness of politicians and scientific advisors tasked with managing the disease have been challenged (Elgar et al., 2020; Public Health England, 2020). While some recent research has found that trust in government is not associated with engagement in preventive behaviours (e.g. Clark et al., 2020), Jaspal et al. (2020) found that trust in politicians was associated with one important preventive behaviour – working from home.

The degree of general trust in advice and guidance from scientific authorities influences both the perceived risk of health hazards (Löfstedt, 2013) and the credibility of specific recommendations for disease prevention (Siegrist et al., 2005). The role of perceived trustworthiness of a source is particularly important when the hazard itself is new and induces fear and panic (Herek et al., 1998). Some studies (e.g. Plohl and Musil, 2020) indicate that greater trust in science and scientists results in a higher estimate of COVID-19 risk because the significance of that risk has been consistently emphasised by the scientific establishment in the UK.

It has been found that people categorised as BAME exhibit higher levels of mistrust of both political and scientific institutions (Kantar, 2019), which may be grounded in long-term perceived discrimination (Combs et al., 2007). Indeed, there is a growing literature on the issue of discrimination and mistrust in relation to healthcare among BAME communities (see Otu et al., 2020). Wellcome (2020) found that 57% of BAME people they sampled reported complete trust or a great deal of trust in information about coronavirus from health scientists, compared with 75% of White people, and 45% of BAME people had either complete trust or a great deal of trust in information from government scientific advisers, compared with 65% of White respondents. Greater BAME mistrust of scientific information may be important because the scientific risk estimates of COVID-19 for BAME people are higher than for White British people. Denial by some BAME people of the trustworthiness of the source of these risk estimates might moderate their estimate of their own risk of COVID-19.

Ingroup power refers to the level of political, economic and cultural influence or control an individual attributes to the category to which they are assigned by society or in which they claim membership. Ingroup power is not a factor typically included in health behaviour models. It is included here specifically because it may affect likelihood of compliance with preventive guidelines by moderating trust in government policy. Trust in those who are in control may be eroded if an individual feels a part of a category that has lower power and less input to decision-making. In some minority groups, there is a well-established belief that they have limited ingroup ‘power’ and control over science, politics and business affairs (Yagmur, 2011). As perceived ingroup power may influence beliefs about one’s own capacity and competence, this also may affect choices about preventive measures.

### Indexing COVID-19 preventive behaviour

Much empirical research into COVID-prevention has focused on specific, or a limited number of, preventive behaviours (e.g. Clark et al., 2020; Harper et al., 2020; Jaspal et al., 2020). In contrast, we use the COVID-19 Preventive Behaviours Index to assess one’s likelihood of engaging in various behaviours (Breakwell et al., 2021). The measure allows us to assess overall perceived likelihood of taking preventive action, rather than focussing upon specific types of behaviour.

### Model predicting COVID-19 preventive behaviour

**Figure 1.**
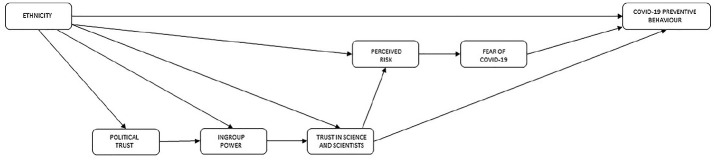
Model predicting COVID-19 preventive behaviour.

Our review of the factors influencing likelihood of COVID-19 preventive behaviour leads to the model presented in Figure 1.

It indicates that BAME and White British people will differ in their likelihood of engaging in COVID-19 preventive behaviours. The model identifies that this occurs through five paths: through a direct path to behaviour and through four other mediated paths. The first mediated path is through levels of political trust, which is then associated with ingroup power and, in turn, with trust in science and scientists. The second path is through a BAME/White British difference in perceived ingroup power that in turn affects trust in science and scientists. The third is through a direct difference between BAME/White British in levels of trust in science and scientists. Trust in science and scientists is directly related to levels of preventive behaviours. It also has a mediated effect on preventive behaviour through perceived own risk of COVID-19, whose influence is in turn mediated by fear of COVID-19. The fourth path is through differences between BAME/White British in perceived own risk of COVID-19. Fear of COVID-19 is directly associated with variation in preventive behaviours. A structural equation model reflecting this theoretical model of direct and mediated effects was tested.

### Hypotheses

Specific hypotheses tested:

1. White British will report higher political trust, trust in science and scientists, ingroup power, and a higher perceived own risk of COVID-19 than BAME people.
2. There will be no significant difference between White British and BAME people in level of fear of COVID-19.
3. Political trust will be positively associated with ingroup power, which is in turn positively associated with trust in science and scientists.
4. Greater trust in science and scientists is associated with greater perceived own risk of COVID-19.
5. Perceived own risk and fear of COVID-19 will be strongly positively associated.
6. Greater fear of COVID-19 and higher trust in science and scientists will be associated with higher likelihood of COVID-19 preventive behaviours.
7. BAME people will be more likely than White British to say they are likely to engage in COVID-19 preventive activity.

We note that the BAME categorisation has been criticised because the term can sometimes blur important differences between the ethnic groups incorporated in it. Consequently, we examined the dataset for evidence of differences within the BAME sample associated with specific ethnic or cultural groupings. These results are also presented.

## Methods

### Ethics

The study received ethics approval from Nottingham University’s College of Business, Law and Social Sciences Ethics Committee. Participants provided electronic consent to participate.

### Participants

A sample of 478 individuals in the United Kingdom was recruited on *Prolific*, an online participant recruitment platform, to participate in a cross-sectional survey study of perceived risk, trust and likelihood of engaging in COVID-19 preventive behaviours. Although a priori power calculations were not performed, following the procedure illustrated by Moshagen and Erdfelder (2016), for RMSEA = 0.06, alpha = 0.05, power = 0.80, and degrees of freedom in the SEM model = 10, we estimated as a satisfactory sample size *N* = 452. Data collection occurred at two points during the pandemic – on 8 July and 14 August 2020. Three hundred and seven participants (64.2%) were female, 169 (35.4%) were male, and 2 (0.4%) were gender non-binary. Participants were aged 18–72 (*M* = 32.7, SD = 12.3) and came from various ethnic and socio-demographic backgrounds. We attempted to recruit a relatively even distribution of White British (*N* = 243, 50.8%) and BAME (*N* = 235, 49.2%) participants for the study, given the empirical focus on differences between these groups. Table 1 includes detailed information on the social and demographic characteristics of participants.

**Table 1.** Socio-demographic characteristics of the sample.

Ethnicity	White British	White Other	White and Black Caribbean	White and Asian	Pakistani	Bangladeshi	Indian	Caribbean	African	Other
	*N* = 243 (50.8%)	*N* = 5 (1%)	*N* = 4 (0.8%)	*N* = 5 (1%)	*N* = 58 (12.1%)	*N* = 16 (3.3%)	*N* = 69 (14.4%)	*N* = 28 (5.9%)	*N* = 48 (10%)	*N* = 2 (0.4%)
Religion	No religion	Christianity	Islam	Hinduism	Sikhism	Judaism	Other			
	*N* = 226 (47.3%)	*N* = 126 (26.4%)	*N* = 66 (13.8%)	*N* = 35 (7.3%)	*N* = 10 (2.1%)	*N* = 2 (0.4%)	*N* = 13 (2.7%)			
Relationship Status	Single	Married	Unmarried – with partner	Cohabiting	Divorced	Civil partnership				
	*N* = 206 (43.1%)	*N* = 138 (28.9%)	*N* = 94 (19.7%)	*N* = 25 (5.2%)	*N* = 11 (2.3%)	*N* = 4 (0.8%)				
Income	Less than £10,000	£10,000–£19,999	£20,000–£29,999	£30,000–£39,999	£40,000–£49,999	£50,000–£59,999	£60,000 or more			
	*N* = 134 (28%)	*N* = 95 (19.9%)	*N* = 112 (23.4%)	*N* = 77 (16.1%)	*N* = 29 (6.1%)	*N* = 9 (1.9%)	*N* = 22 (4.6%)			
Employment status	Employed	Self-employed	Furloughed	Student	Retired	Unemployed				
	*N* = 243 (50.8%)	*N* = 37 (7.7%)	*N* = 32 (6.7%)	*N* = 114 (23.8%)	*N* = 10 (2.1%)	*N* = 42 (8.8%)				
Education	Undergraduate Degree	A-/AS-Levels	GCSE/O Level	Postgraduate Degree	Apprenticeship	Other	None			
	*N* = 199 (41.6%)	*N* = 141 (29.5%)	*N* = 49 (10.3%)	*N* = 76 (15.9%)	*N* = 45 (1%)	*N* = 1.5 (2%)	*N* = 1 (0.2%)			
Gender	Male	Female	Non-binary							
	*N* = 169 (35.4%)	*N* = 307 (64.2%)	*N* = 2 (0.4%)							

### Measures

All measures were computed using averages after scale reliability was deemed acceptable.

#### Political trust

The Political Trust Questionnaire (Mutz and Reeves, 2005) was adapted to measure political trust specifically in the context of COVID-19. The adapted scale consisted of four items, such as ‘Politicians generally have good intentions in relation to COVID-19’ and ‘Politicians can be trusted to do what is right in relation to COVID-19’. The items were measured on a 5-point scale (1 = strongly disagree to 5 = strongly agree) (α = 0.87; *M* = 10.44; SD = 4.44).

#### Trust in science and scientists

The Trust in Science and Scientists Inventory (Nadelson et al., 2014) was used to measure trust in science and scientists. The original scale consisted of 21 items, measured on a 5-point scale. A higher score indicated greater trust in science and scientists. We performed exploratory and confirmatory factor analyses on the scale and identified a multidimensional structure (with three factors). The first factor (comprising 12 items) accounted for items of theoretical interest in the current study, such as ‘Scientists ignore evidence that contradicts their work.’ and ‘Scientific theories are weak explanations’. We used these items in our subsequent analyses (α = 0.89; *M* = 41.26; SD = 6.49). Details of the factor analyses are included in Appendix 1.

#### Ingroup power

Six items were adapted from the Subjective Vitality Questionnaire (Bourhis et al., 1981) to measure perceived ingroup power of White British people and for BAME people in the UK. Items included ‘How much political power do White British/ BAME people have in the UK? and ‘How much control do White British/ BAME people have over economic and business matters in the UK?’ Items were measured on a 5-point scale (1 = not at all well to 5 = extremely well). The variable of ingroup power was created by calculating a composite score for White British people’s perception of White British people’s power and BAME people’s perception of BAME people’s power. White British participants responded to ‘White British people’s power’ items only, and BAME participants responded to ‘BAME people’s power’ items only. Cronbach’s alpha for the scale was 0.84, *M* = 19.64, SD = 7.11.

#### Fear of COVID-19

The Fear of COVID-19 Scale (Ahorsu et al., 2020) was used, but adapted to avoid response bias in phrasing. The adapted scale included 10 items and was measured on a 5-point scale (1 = strongly disagree to 5 = strongly agree). Items included ‘I do not worry much about COVID-19’ and ‘When I think about COVID-19, my heart races and palpitates’. A higher score indicated greater fear of COVID-19 (α = 0.83; *M* = 24.77; SD = 5.51).

#### Perceived own risk of COVID-19

The COVID-19 Own Risk Appraisal Scale (CORAS) (Jaspal et al., 2020) was used to measure one’s own perceived risk of exposure to COVID-19. The scale consisted of six items and items were measured a 5-point scale (1 = strongly disagree to 5 = strongly agree). Items included: ‘I am sure I will NOT get infected with COVID-19’ and ‘I feel vulnerable to COVID-19 infection’. A higher score indicated higher perceived own risk of COVID-19 (α = 0.85; *M* = 17.93; SD = 4.44).

#### COVID-19 preventive behaviours

The COVID-19 Preventive Behaviours Index (Breakwell et al., 2021) was used to measure the likelihood of engaging in specific behaviours that can decrease one’s risk of coronavirus infection. The scale consisted of 10 items, which were measured on a 5-point scale (1 = extremely unlikely to 5 = extremely likely). Items included ‘How likely is it that, during the COVID-19 outbreak you will keep a distance of 2 metres in your everyday interactions with people outside of your household?’ and ‘. . .avoid any non-essential local travel?’ A higher score indicated greater COVID-19 preventive behaviours (α = 0.78; *M* = 36.04; SD = 5.57).

#### Ethnicity

In addition to the participants’ categorisation as White British, Black South Asian or Black British, we produced a binary variable including two groups: White British individuals (0) and BAME (1) individuals.

### Data analysis strategy

We used one-way ANOVA to test mean differences between ethnic groups in all the variables in our theoretical model, with pairwise comparisons with Holm-Bonferroni corrections. We estimated Pearson’s product-moment correlation coefficients across all the variables in the model, overall and split by three ethnic groups.

We fitted, evaluated, and compared a series of alternative structural equation models (SEMs) aiming to investigate the role of the variables and their different relationships. We used maximum likelihood estimation with no imputation methods, given the absence of missing data. The following fit indices and criteria were used to evaluate the goodness of fit: The Chi-Squared test of goodness of fit, accepting a ratio of the Chi-Squared estimate to degrees of freedom <3 as acceptable (Kline, 2011); The Comparative Fit Index (CFI) ⩾ 0.95, the Tucker Lewis Index (TLI) ⩾ 0.95, the Root Mean Square Error of Approximation (RMSEA) ⩽ 0.07, the Standardized Root Mean Square Residual < 0.08 (Brown, 2006).

We used differences in CFI and RMSEA to compare SEMs, considering a decrease in CFI > 0.09 and an increase in RMEA > 0.14 as indicative of worse fit (Schumacker and Lomax, 2016). Before running the models, we checked for the possible multicollinearity of the variables that we used as predictors in the model, using COVID-19 preventive behaviour as the outcome variable, predicted by all other variables in the model. We considered values of Variance Inflation Factor (VIF) >5 as indicative of multicollinearity (James et al., 2014).

We estimated indirect effects in SEM by means of bootstrapping (1,000 repetitions), and we considered paths as statistically significant if the bootstrapped confidence intervals did not contain zero (Kenny, 2018).

All analyses were performed by means of the statistical programming language R, and in particular, the packages lavaan (Rosseel, 2012) and semTools (Jorgensen et al., 2020).

### Data sharing statement

The datafile containing all of the variables analysed in this study is in the Supplemental Material section of the Journal website.

## Results

### Descriptive statistics

Table 2 provides descriptive statistics for the variables included in the theoretical model separately for White British, British South Asians, and Black British. It includes the results of the one-way ANOVA which showed there were significant one-way differences between the three groups on all the variables except COVID-19 Preventive Behaviours.

**Table 2.** Descriptive statistics for ethnic differences for key variables of interest and results of One-Way ANOVAs.

	White British			British South Asians			Black British			*F*	df	*p*	η^2^
	*N*	*M*	SD	*N*	*M*	SD	*N*	*M*	SD				
Political trust	253	2.75	0.95	173	2.45	0.86	52	2.49	0.83	6.20	2, 475	<0.003	0.03
Trust in science and scientists	253	3.54	0.51	173	3.34	0.57	52	3.25	0.48	11.37	2, 475	<0.001	0.05
Perceived ingroup power	253	4.17	0.71	173	2.32	0.73	52	2.11	0.64	426.20	2, 475	<0.001	0.64
Fear of COVID-19	253	2.85	0.65	173	2.78	0.66	52	2.8	0.65	0.66	2, 475	0.05	0.00
Perceived own risk of COVID-19	253	3.07	0.72	173	2.96	0.77	52	2.69	0.69	6.24	2, 475	<0.001	0.03
COVID-19 preventive behaviours	253	3.95	0.63	173	4.08	0.6	52	3.96	0.65	2.45	2, 475	0.09	0.01

Post-hoc analyses showed there were statistically significant differences between the White British and the British South Asian groups in: political trust (*p* < 0.003), trust in science and scientists (*p* < 0.001), and ingroup power (*p* < 0.001); with White British reporting higher ratings on each of these variables. There were statistically significant differences between the White British and the Black British groups in: trust in science and scientists (*p* < 0.001), ingroup power (*p* < 0.001), and perceived risk of COVID-19 (*p* < 0.001); with the White British reporting higher ratings on each of these variables. Black British and British South Asian groups differed significantly in perceived own risk of COVID-19 (*p* < 0.05); with British South Asians rating their risk higher.

### Correlations between the variables in the theoretical model

Pearson’s product-moment correlations indicated that political trust was positively associated with ingroup power; that trust in science and scientists was positively associated with ingroup power and with COVID-19 preventive behaviours; and that fear of COVID-19 and perceived own risk of COVID-19 were both positively associated with COVID-19 preventive behaviours. Table 3 presents the correlations between the variables for the whole sample and broken down by ethnic group. There are notable differences between ethnic groups. For White British and Black British trust in politics is not significantly related to other variables but for South Asian British it is significantly positively associated with ingroup power. Trust in science and scientists is positively correlated with ingroup power and COVID-19 preventive behaviours for White British and British South Asian British but not Black British. Fear of COVID-19 is positively related to COVID-19 risk in all groups and with COVID-19 preventive behaviours for White British and South Asian British but not for Black British.

**Table 3.** Correlation matrix of key variables of interest, overall and by ethnic groups.

Variables	1	2	3	4	5
Overall					
1. Trust in politics					
2. Trust in science and scientists	0.05				
3. Perceived ingroup power	0.21**	0.32**			
4. Fear of COVID-19	−0.05	−0.04	0.02		
5. Perceived own risk of COVID-19	−0.06	0.12	0.08	0.55**	
6. COVID-19 preventive behaviours	0.02	0.26**	−0.02	0.27**	0.22**
White British					
1. Trust in politics					
2. Trust in science and scientists	0.00				
3. Perceived ingroup power	−0.12	0.31**			
4. Fear of COVID-19	−0.02	0.01	0.00		
5. Perceived own risk of COVID-19	−0.06	0.14	−0.01	0.57**	
6. COVID-19 preventive behaviours	0.01	0.31**	0.12	0.33**	0.28**
South Asian British					
1. Trust in politics					
2. Trust in science and scientists	0.08				
3. Perceived ingroup power	0.51**	0.23**			
4. Fear of COVID-19	−0.14	−0.14	−0.11		
5. Perceived own risk of COVID-19	−0.11	0.04	−0.07	0.53**	
6. COVID-19 preventive behaviours	0.05	0.28**	0.11	0.22**	0.20**
Black British					
1. Trust in politics					
2. Trust in science and scientists	−0.10				
3. Perceived ingroup power	0.32	−0.12			
4. Fear of COVID-19	−0.06	0.02	−0.15		
5. Perceived own risk of COVID-19	−0.10	0.04	−0.06	0.48**	
6. COVID-19 preventive behaviours	0.11	0.14	−0.17	0.19	−0.01

** *p* < 0.001.

### Structural equation model

All independent variables showed acceptable values of VIF (ethnicity = 3.06, trust in politics = 1.06, ingroup power = 3.32, trust in science and scientists = 1.15, perceived own risk of COVID-19 = 1.51, fear of COVID-19 = 1.47).

We ran, evaluated, and compared a series of alternative SEMs: (1) A baseline model, with all the hypothesised patterns specified; (2) a model with political trust and trust in science and scientists in opposite order, compared to the baseline model; (3) a model nested within Model 1, obtained by constraining the effect of ethnicity to zero; (4) a model nested within Model 2, obtained by constraining the effect of ethnicity to zero; (5) a model nested within Model 1, obtained by constraining the effect of ingroup power to zero; (6) a model nested within Model 2, obtained by constraining the effect of ingroup power to zero.

As hypothesised, the baseline model had excellent fit to the data. Moreover, all nested models showed large decreases in CFI and large increases in RMSEA, indicating a loss of model fit resulting from constraining to zero those paths, and highlighting the key role of differences in ethnicity and ingroup power in explaining preventive behaviour in the model (Table 4).

**Table 4.** Structural models, fit indices.

Model number	Model description	CFI	RMSEA	SRMR
1	Baseline model	0.994	0.034	0.032
2	Trust in Politics and Trust in Science and Scientists in inverted positions	0.942	0.104	0.059
3	Model 1 after constraining Ethnicity to zero	0.382	0.287	0.167
4	Model 1 after constraining Ingroup Power to zero	0.341	0.296	0.174
5	Model 2 after constraining Ethnicity to zero	0.956	0.086	0.044
6	Model 2 after constraining Ingroup Power to zero	0.927	0.111	0.063

**Figure 2.**
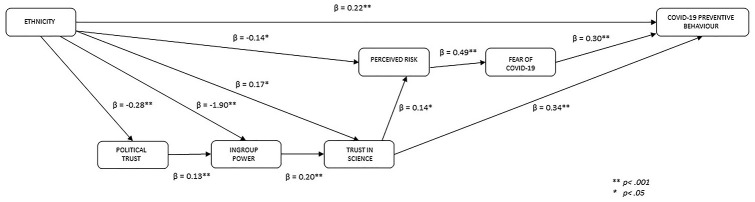
Direct paths between variables in model predicting COVID-19 preventive behaviour (For SEs and CIs see Table 5).

Finally, we estimated and interpreted direct, indirect and total effects, using 1,000 bootstrap repetitions. Table 5 presents a summary of the effects in the model and Figure 2 illustrates the direct paths between variables. Unstandardised betas are reported throughout.

**Table 5.** Baseline model: Effects and standard errors (1000 bootstrap repetitions).

Effects	β	SE	*p*	95% CI – Lower	95% CI – Upper
Direct effects					
Being BAME > Trust in politics	−0.28	0.08	<0.001	−0.24	−0.07
Being BAME > Perceived ingroup power	−1.90	0.06	<0.001	−0.83	−0.77
Trust in politics > Perceived ingroup power	0.13	0.03	<0.001	0.05	0.15
Being BAME > Trust in science and scientists	0.17	0.08	<0.05	0.01	0.30
Perceived ingroup power > Trust in science and scientists	0.20	0.03	<0.001	0.30	0.59
Trust in science > Perceived own risk of COVID-19	0.14	0.07	<0.05	0.01	0.20
Being BAME > Perceived own risk of COVID-19	−0.14	0.07	<0.05	−0.19	0.00
Perceived own risk of COVID-19 > Perceived fear of COVID-19	0.49	0.04	<0.001	0.48	0.63
Trust in science and scientists > COVID-19 preventive behaviour	0.34	0.05	<0.001	0.20	0.37
Being BAME > COVID-19 preventive behaviour	0.22	0.05	<0.001	0.10	0.26
Perceived fear of COVID-19 > COVID-19 preventive behaviour	0.30	0.04	<0.001	0.23	0.39
Indirect effects					
Being BAME > Trust in politics > Perceived ingroup power	0.53	0.16	<0.001	0.05	0.19
Being BAME > Perceived ingroup power > Trust in science and scientists	−0.39	0.07	<0.001	−0.47	−0.24
Being BAME > Trust in politics > Perceived ingroup power > Trust in science and scientists	−0.01	0.00	<0.03	−0.01	0.00
Being BAME > Trust in science and scientists > Perceived own risk of COVID-19	0.02	0.02	0.17	0.00	0.04
Being BAME > Trust in politics > Perceived ingroup power > Trust in science and scientists > Perceived own risk of COVID-19	0.00	0.00	0.15	0.00	0.00
Being BAME > Trust in science and scientists > COVID-19 preventive behaviour	0.08	0.04	<0.05	0.01	0.17
Being BAME > Perceived ingroup power > Trust in science and scientists > COVID-19 preventive behaviour	−0.19	0.04	<0.001	−0.28	−0.13
Being BAME > Trust in politics > Perceived ingroup power > Trust in science and scientists > COVID-19 preventive behaviour	0.00	0.00	<0.04	−0.01	0.00
Being BAME > Trust in science and scientists > Perceived own risk of COVID-19 > Fear of COVID-19 > COVID-19 preventive behaviour	0.00	0.00	0.22	0.00	0.00
Being BAME > Perceived ingroup power > Trust in science and scientists > Perceived own risk of COVID-19 > Fear of COVID-19 > COVID-19 preventive behaviour	−0.01	0.00	0.10	−0.01	0.00
Being BAME > Trust in politics > Perceived ingroup power > Trust in science and scientists > Perceived own risk of COVID-19 > Fear of COVID-19 > COVID-19 preventive behaviour	0.00	0.00	0.19	0.00	0.00
Total effects					
Being BAME > Trust in politics	0.66	0.16	<0.001	0.14	0.31
Being BAME > Trust in science and scientists	−0.22	0.05	<0.001	−0.29	−0.12
Being BAME > Perceived risk of COVID-19	−0.12	0.08	0.11	−0.18	0.02
Being BAME > COVID-19 preventive behaviour	0.11	0.06	0.07	−0.03	0.15

Results showed that being BAME was significantly associated with lower trust in politics; lower ingroup power; higher trust in science and scientists; lower perceived own risk of COVID-19; and higher COVID-19 preventive behaviour. The effect of being BAME on ingroup power was partially mediated by trust in politics. Also, the effect of being BAME on trust in science and scientists was partially mediated by ingroup power and by the indirect effect of ingroup power on COVID-19 preventive behaviour. The effect of being BAME on COVID-19 preventive behaviour was mediated by trust in science and scientists.

The serial indirect effects that were found supported the theoretical model proposed in Figure 1. Higher trust in politics was significantly associated with higher ingroup power, which in turn was associated significantly with higher trust in science and scientists. Higher trust in science and scientists was significantly associated with higher perceived own risk of COVID-19 and higher COVID-19 preventive behaviour. Higher perceived own risk of COVID-19 was significantly associated with higher perceived fear of COVID-19. Higher perceived fear of COVID-19 was significantly associated with higher COVID-19 preventive behaviour.

Contrary to our hypothesis, in the SEM being BAME was positively associated with trust in science and scientists. Following the procedure illustrated by Watson et al. (2013), we investigated the possible suppression effect produced when transitioning from a model accounting for ethnicity alone to a model in which ethnicity and ingroup power jointly predicted trust in science and scientists. We first analysed the effect of ethnicity on trust in science and scientists alone, and then the effect of ethnicity after adding ingroup power, by means of simple and multiple linear regression analyses, respectively. The results showed a substantial suppression effect, with the association between ethnicity alone (β = 0.21, SE = 0.02, *p* < 0.001) and trust in science and scientists shifting from positive to negative (β = −0.16, SE = 0.04, *p* < 0.05) when adding ingroup power (β = 0.45, SE = 0.03, *p* < 0.001) in the model, with an increment in adjusted R-Squared from 0.04 to 0.11, respectively. Results from the Sobel test showed that the suppression effect was statistically significant (z = 5.88, *p* < 0.001).

## Discussion

Our results show differences between responses of White British and BAME people to factors that shape their reactions to COVID-19. It particularly highlights the significance of the perception of ingroup power. Notwithstanding the diversity within the BAME category, it has been established as a societally recognised conceptual group. The discrepancy in the reported perceptions of ingroup power among White British and BAME people constitutes an important indicator that individuals hold strong social representations of the relative social status of their own category. Ingroup power was defined in terms of control and competence across a broad spectrum of activities (including, politics, the economy and business, the mass media, culture and the arts). Crucially, in our sample, BAME respondents perceived the BAME conceptual group as having less ingroup power.

### Model of the influences upon COVID-19 preventive behaviours

Our SEM analysis generally supports the model of the direct and mediated effects of BAME/White British upon COVID-19 preventive behaviours predicted in Figure 1. The findings entail three elements. First, perceived personal risk of COVID-19 infection and fear of COVID-19 were strongly associated and fear of the disease predicts COVID-19 preventive behaviours (see also Khosravi, 2020). Second, higher trust in science and scientists was associated with greater perceived personal risk. Trust in science and scientists was associated directly with greater likelihood of taking preventive measures and, also, through its impact on risk perception. Third, ethnicity (being White British or BAME) had an impact on levels of trust both in science and scientists and in politicians, with BAME people in the sample generally reporting less trust in both. However, in the SEM, when the effects of perceived ingroup power were taken into consideration, being BAME appeared to be associated with a higher level of trust in science and scientists. This finding can be attributed to the suppression effect when ingroup power was added in the model (see Watson et al., 2013), suggesting the need for further investigation of perceptions of ingroup power as determinants of preventative and precautionary health behaviour. Indeed, political trust was positively related to ingroup power. BAME also reported lower ingroup power. The relationship between ingroup power and trust in science and scientists is particularly notable. The higher the perceived power of the ingroup, the greater the trust in science and scientists. Through this route, ingroup power helps to predict COVID-19 preventive behaviours. Given the considerable disparity between White British and BAME people in their perception of the power of their ingroups, this channel of influence on preventive behaviours is important.

### Trust in science and scientists

Public trust in science has changed during the COVID-19 pandemic (Agley, 2020). In our study trust in science and scientists facilitated the likelihood of engaging in preventive behaviours directly as well as indirectly through its impact on perceived personal risk. It is notable that this trust predicts preventive activity at a time when a high-risk message about COVID-19 and recommendations for significant, often disliked, behavioural changes were coming from the scientific establishment. Simultaneously, much conspiracy theorising in relation to COVID-19 focused on the de-legitimisation of science and scientists (Jaspal et al., 2013), questioning both their competence and motives. Inculcating mistrust in such authorities is the basis for redirecting, if not controlling, behavioural change. Perceived ingroup power appears to diminish the potency of such attacks on the trustworthiness of science and scientists (Krause et al., 2019). This could be explained by the perceived efficacy of more powerful groups in influencing and participating in the scientific community and benefiting from it. Indeed, we found that the perception of ingroup powerlessness was associated with mistrust of science and scientists as well as politicians. Perhaps this is not surprising – feeling that you have little control over someone or something tends to be associated with doubt, suspicion and uncertainty (Ross et al., 2001).

### BAME likelihood of preventive behaviour

In addition to the indirect effects of ethnicity through the other variables examined, the model highlights that ethnic category has a direct path to COVID-19 preventive behaviours. BAME people reported they were more likely to adopt the 10 preventive behaviours they rated than did the White British. This reflects an underlying pattern in the results: compared to White British participants in the sample, BAME people’s reported likelihood of preventive behaviour was less strongly linked to trust in science or scientists, perceived personal risk of COVID-19 or fear of it.

Some additional factor is needed to account for the fact that BAME people report greater likelihood that they will participate in preventive behaviours. Their likelihood of adopting preventive behaviours may be particularly affected by the epidemiological data showing risk of coronavirus infection and severity of consequences to be greater in the BAME conceptual group (Pan et al., 2020). Reports of this greater risk to their ingroup were well-publicised in the national media and government briefings. However, it is evident that this differential between objective levels of group risk did not become reflected in assessments of perceived personal risk. This disparity in personal- and group-level risk perceptions has been variously explained in the past by reference to subjective immunity, perceived invulnerability or optimistic bias (Asif et al., 2020; Park et al., 2020). Faced with clear objective evidence of high ingroup risk, individuals typically will rate their own risk as less than the risk of the average person. Perceived ingroup risk was not measured in this study but it is possible that higher perceived ingroup risk resulted in greater willingness to engage in preventive behaviours. Personal preventive behaviour may then be explained by a desire to protect others, as well as oneself, over and above any concern derived from perceived own risk. Altruism may be a basis for following prevention guidance.

While our study emphasises the importance of examining the reasons for differences between BAME and White British responses to COVID-19 preventive behaviour, it also suggests examining further differences within the BAME conceptual group would be valuable. Our study provides preliminary evidence of significant differences between the two main constituent parts of the BAME conceptual group (British South Asian vs Black British people) on several key variables related to COVID-19 preventive activity. On average, British South Asians reported much higher levels of trust in science and scientists, ingroup power, perceived own risk of COVID-19 and COVID-19 preventive activity than Black British people. Future research is needed to test the replicability of these findings. However, our findings suggest that it would be beneficial to develop interventions to build confidence in science and scientists and for effective risk communication within Black British communities within the BAME conceptual group, in particular. Moreover, efforts to increase perceived ingroup power among Black British people are likely to enhance trust and, thereby, raise COVID-19 own risk perception.

### Future directions

Subsequent research should use methods additional to the online survey to collect data. The online survey method may bias sampling (indirectly excluding the more difficult to access groups, for instance those with certain disabilities, lower education or those with limited access to digital technology). However, it is also important not to ignore the speed and scale of data available online and the benefits of this form of data collection when dealing with a fast-moving societal phenomenon like COVID-19.

Given that the pandemic itself is morphing rapidly over time, social science research needs to focus on capturing systematically changes in behavioural responses to it. This should include short-interval, cohort sequential and longitudinal measurements of actual behaviours as well as self-reports of behaviour or intentions about, or perceived likelihood of, behaviour.

The theoretical model we presented is a good fit based on the variables we measured but other variables, such as perceived ingroup risk and altruistic motives, need to be examined further in additional samples if a more comprehensive explanation of COVID-19 preventive behaviour is to be developed. Indeed, the significance of other predictors of adherence to guidelines on prevention, such as self-efficacy (Bogg and Milad, 2020) and personal beliefs (Lees et al., 2020) have already been mooted.

## Conclusion

This study represents a snapshot at one period of the pandemic, in one country, in the midst of changing guidance on preventive measures. However, as indicated in the introduction, the model presented builds on earlier studies of the social psychological precursors to preventive behaviour. This model is also explicitly different from earlier work in emphasising and testing the role of perceived ingroup power in predicting likelihood of preventive behaviour. Practical recommendations derived from our study would include the promotion of greater trust in science and scientists that may be achieved more easily if people perceive that their ingroup is engaged with the scientific community. This engagement should aim at fostering a sense of public ownership of science, responsibility for it and respect of it.

## Research Data

sj-sav-1-hpq-10.1177_13591053211017208 – COVID-19 preventive behaviours in White British and Black, Asian and Minorty Ethnic (BAME) people in the UK Click here for additional data file. sj-sav-1-hpq-10.1177_13591053211017208 for COVID-19 preventive behaviours in White British and Black, Asian and Minorty Ethnic (BAME) people in the UK by Glynis M Breakwell, Emanuele Fino and Rusi Jaspal in Journal of Health Psychology This article is distributed under the terms of the Creative Commons Attribution 4.0 License ( https://creativecommons.org/licenses/by/4.0/) which permits any use, reproduction and distribution of the work without further permission provided the original work is attributed as specified on the SAGE and Open Access pages ( https://us.sagepub.com/en-us/nam/open-access-at-sage).

sj-pdf-2-hpq-10.1177_13591053211017208 – Supplemental material for COVID-19 preventive behaviours in White British and Black, Asian and Minorty Ethnic (BAME) people in the UK Click here for additional data file. Supplemental material, sj-pdf-2-hpq-10.1177_13591053211017208 for COVID-19 preventive behaviours in White British and Black, Asian and Minorty Ethnic (BAME) people in the UK by Glynis M Breakwell, Emanuele Fino and Rusi Jaspal in Journal of Health Psychology
