# Supplementary material for: COVID-19 preventive behaviours in White British and Black, Asian and Minority Ethnic (BAME) people in the UK
Source: J Health Psychol. 2021 May 15;27(6):1301–17. doi: 10.1177/13591053211017208 (PMC9036160; doi:10.1177/13591053211017208)
Supplement: sj-pdf-2-hpq-10.1177_13591053211017208 – Supplemental material for COVID-19 preventive behaviours in White British and Black, Asian and Minorty Ethnic (BAME) people in the UK [file sj-pdf-2-hpq-10.1177_13591053211017208.pdf]

Appendix 1: Factor structure and psychometric properties of the Trust in Science and Scientists Inventory

Results from exploratory factor analysis showed that a three-factor model was the best fit to the data, with the pattern matrix displaying 12 items loading onto the first factor, 4 items loading onto the second factor, and 4 items loading onto the third factor, and no cross-loadings (Figure 1).

Figure 1 here

In particular, the first factor accounted for items of theoretical interests in the current study, such as ‘Scientists ignore evidence that contradicts their work.’ and ‘Scientific theories are weak explanations.’. For this reason, we decided to focus on the first sub-scale, including a total of 12 items. We tested the properties of the sub-scale by means of confirmatory factor analysis, testing a model with 12 items loading onto a single latent dimension. Results showed acceptable fit (CFI = 0.953, RMSEA = 0.063 [90% CI = 0.051-0.074], SRMR = 0.037). The sub-scale was internally consistent (Cronbach’s alpha = 0.89). Based on such evidence, we retained and utilised the 12-items sub-scale in subsequent analyses.

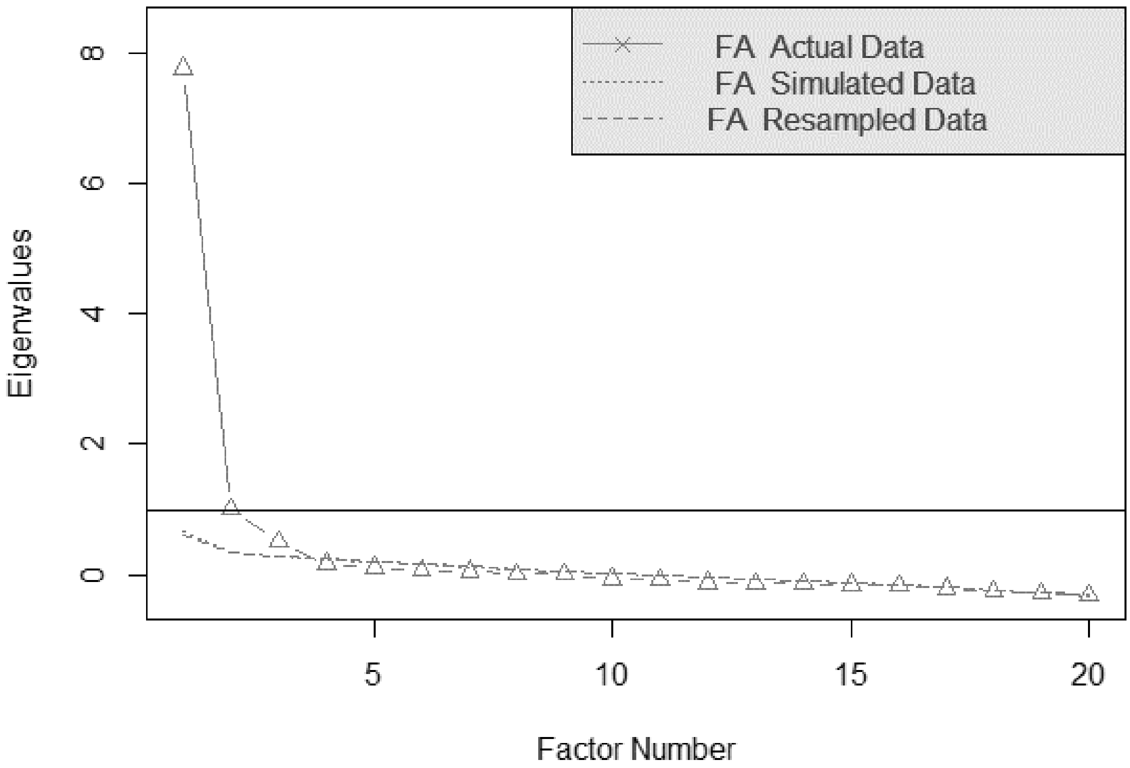

Figure 1: Trust in Science and Scientists Scale, parallel analysis.
